# Understanding the Relationship between Socio-Economic Status, Physical Activity and Sedentary Behaviour, and Adiposity in Young Adult South African Women Using Structural Equation Modelling

**DOI:** 10.3390/ijerph14101271

**Published:** 2017-10-23

**Authors:** Lisa K. Micklesfield, Richard J. Munthali, Alessandra Prioreschi, Rihlat Said-Mohamed, Alastair van Heerden, Stephen Tollman, Kathleen Kahn, David Dunger, Shane A. Norris

**Affiliations:** 1 MRC/Wits Developmental Pathways for Health Research Unit, Department of Paediatrics, School of Clinical Medicine, Faculty of Health Sciences, University of the Witwatersrand, Johannesburg 2193, South Africa; munthalirichard@gmail.com (R.J.M.); Alessandra.prioreschi@wits.ac.za (A.P.); rihlat.saidmohamed@wits.ac.za (R.S.-M.); avanheerden@hsrc.ac.za (A.v.H.); san@global.co.za (S.A.N.); 2 Human and Social Development Research Programme, Human Sciences Research Council, 22 Mbuvu Dr, Sweetwater, Pietermaritzburg 3201, South Africa; 3 MRC/Wits Rural Public Health and Health Transitions Research Unit (Agincourt), School of Public Health, Faculty of Health Sciences, University of the Witwatersrand, Johannesburg 2193, South Africa; stephen.tollman@wits.ac.za (S.T.); kathleen.kahn@wits.ac.za (K.K.); 4 Epidemiology and Global Health Unit, Department of Public Health and Clinical Medicine, Umeå University, SE-901 87 Umeå, Sweden; 5 INDEPTH Network, 38 & 40 Mensah Wood Street, East Legon, Accra, Ghana; 6 Department of Paediatrics, University of Cambridge and Wellcome Trust-MRC Institute of Metabolic Science, Cambridge CB2 0QQ, UK; dbd25@cam.ac.uk

**Keywords:** socio-economic status, urban, rural, physical activity, South Africa, sedentary, body mass index, waist circumference

## Abstract

Socio-economic status (SES) is an important predictor of obesity, but how it is associated with differences in physical activity and sedentary behaviour is less clear. This cross-sectional study examined the association between SES (sum of household assets), physical activity and sedentary time, and how they predict adiposity. Socio-demographic, anthropometric, and physical activity data on rural (*n* = 509) and urban (*n* = 510) South African women (18–23 years) were collected. Overweight and obesity prevalence, and sedentary time, were higher; and moderate-vigorous intensity physical activity (MVPA) was lower, in the urban sample. Structural equation models (SEMs) were constructed for BMI and waist circumference. In the urban sample SES had a direct inverse effect on MVPA (ß; 95% CI, −41.69; −73.40 to −9.98), while in the rural sample SES had a direct effect on BMI (ß; 95% CI, 0.306; 0.03 to 0.59). In the pooled sample, SES had a direct inverse effect on MVPA (ß; 95% CI, −144; −170.34 to −119.04), and MVPA was directly associated with BMI (ß; 95% CI, 0.04; 0.01 to 0.08). The influence of SES, and the role of physical activity and sedentary time on adiposity differs between the urban and rural samples, and the importance of other environmental and behavioural factors must be considered in the development of obesity and the design of effective interventions.

## 1. Introduction

Socio-economic status (SES) has been well described as an important determinant of physical activity and sedentary behaviour, however, these associations are not consistent across the literature, particularly in adolescents [1]. We have previously shown in a sample of rural South African adolescents that SES at the maternal, household, and community levels independently predicted time spent in sedentary behaviours, as well as time spent participating in school and club moderate to vigorous intensity physical activities [2]. Further, the association between SES and body mass index (BMI) differs between high-income and low- or middle-income countries, with studies from high income countries reporting an inverse association between SES and obesity [3,4] and studies from many sub-Saharan African countries showing a positive association between SES and obesity [5,6,7,8]. National data from South Africa has shown that this association may not be linear [9], and may be influenced by different physical activity patterns across communities, as well as other factors, such as food security and access to nutrition.

Results from the 2016 Healthy Active Kids South Africa (HAKSA) report card [10] have shown that although more than 50% of South African children and adolescents are meeting physical activity recommendations, physical activity participation is lower in girls than boys, and decreases with age. Active travel is still a significant contributor to daily physical activity in South African children and adolescents, and has recently been shown to be high in rural adolescents [2] in whom the prevalence of overweight and obesity is also increasing [11]. Dugas et al. [12] have recently shown in each of their study sites from five low-and middle-income countries, including South Africa, that there was a significantly greater increase in body weight over a two year period in participants meeting physical activity guidelines compared to those who did not meet the guidelines. This suggests that the traditionally-accepted inverse association between physical activity and BMI may be confounded by changing food environments in transitioning societies, and that other factors may play a more significant role in determining BMI and body composition. Time spent in sedentary behaviours has also been shown to be high in some South African populations [13,14], but whether this predicts adiposity in the context of socio-demographic factors in urban and rural populations remains to be investigated. Further, a study of young adult women from urban and rural settings in South Africa is of great relevance as they fall within an age group that has been reported to have the highest pregnancy rates in South Africa [15]. Recent data indicate that 26.2% of births in South Africa between 2013 and 2015 occurred in females aged 20–24 years, with the next highest prevalence in females aged 25–29 years (25.7%). Understanding factors associated with overweight and obesity in this population will have important implications for their risk of disease [16], as well as that of their offspring [17]. The aim of this study is, therefore, to examine the association between SES, physical activity and sedentary time, and how they determine whole body and central adiposity in young adult South African women living in either urban or rural settings.

## 2. Materials and Methods

### 2.1. Study Sample and Design

The rural sample included in this cross-sectional study consisted of female participants (*n* = 509) aged between 18–21 years old who were selected from the census data of the Agincourt Health and Socio-Demographic Surveillance System (Agincourt HDSS, 475 km^2^, 31 villages, 110,000 inhabitants) [18]. Of the *n* = 2126 eligible females, *n* = 996 were located and invited by telephone or by visiting their home, to participate in the study. Data was collected on *n* = 509 with reasons for non-participation including refusals (*n* = 249), death, and moved away (*n* = 115), did not attend appointment (*n* = 112), and inconsistencies with census data (*n* = 11). In the urban site (Soweto, Johannesburg, 200.03 km^2^, over 1,271,628 inhabitants), 510 young adult women were randomly selected from the Birth to Twenty cohort study which is the largest and longest running longitudinal cohort in Africa [19]. The original birth cohort was recruited in 1990 in order to understand child development and health at a time when it was expected that there would be a greater demand for health care services in urban areas due to urbanization. A total of 3273 study participants were enrolled in the birth cohort from 23 April to 8 June 1990 and the original cohort was representative of the South African racial demographics. The urban cohort in this study represents a random sub-sample of the 720 women recruited for follow up at the first data collection time point after the age of 20 years, referred to as the Young Adult Survey, and only includes the black African women who were part of the original cohort. All pregnant women (*n* = 51) were excluded. All participants provided written consent to participate in the studies. The Human Research Ethics Committee of the University of the Witwatersrand (clearance certificates M120138 for the Ntshembo-Hope Cross-Sectional Survey and M111182 for the BT20+ survey) approved the study protocols.

### 2.2. Anthropometrics

Anthropometry was measured by trained field workers and included: weight measured in light clothing and barefoot to the nearest 0.1 kg using a digital scale (Tanita model TBF-410; Arlinghton Heights, IL, USA); height measured barefoot to the nearest 0.1 cm using a wall mounted stadiometer (Holtain, Crymych, UK); and waist and hip circumferences measured with a non-stretchable fibreglass insertion tape at the level of the umbilicus, and at the largest gluteal diameter, respectively. BMI was calculated as the weight/height^2^ in kg/m^2^.

### 2.3. Socio-Economic Status (SES)

Questionnaires were completed via interview by trained field workers. A household SES index was generated by summing the number of assets owned in the household from the following: TV, car, washing machine, fridge, phone, radio, microwave, cell phone, DVD/Video, DSTV (cable channel), computer, internet, medical aid (private medical insurance). This index has been described as a useful method for determining socio-economic status [20]. All girls were required to report the highest grade that they had successfully completed, and this was then categorised into primary, secondary, and tertiary education.

### 2.4. Physical Activity and Sedentary Behaviour

The Global Physical Activity Questionnaire (GPAQ), developed for global physical activity surveillance, was completed via interview to obtain self-reported physical activity [21]. Total moderate-vigorous intensity physical activity (MVPA) in minutes per week (minutes/week) were calculated by adding occupation, travel-related and leisure time moderate and vigorous intensity physical activity. Sitting time (minutes/week) was used as a proxy for sedentary behaviour. 

### 2.5. Statistical Analyses

Analysis of variance, Student’s *t*-test, and chi-squared tests were completed to compare differences in study characteristics between urban and rural young women. Structural equation modelling (SEM) was used to test and estimate the relationship between variables, specifically the mediation role of physical activity (MVPA) and sedentary behaviour (sitting) on the association between SES and body composition (BMI and waist circumference). The structural equation consists of two parts, the structural model and the measurement model. The structural model defines the relationship between the composite latent variables and other observed variables, while the measurement model represents the relationship between measured and composite latent variables. Direct, indirect, and total effects were computed and recoded, and the proportion of the total effect mediated was calculated.

To evaluate the best fitting model for our data, we calculated and recorded different goodness of fit indices including chi-squared test, Root mean squared error of approximation (RMSEA), comparative fit index (CFI), Tucker-Lewis index (TLI), and standardized root mean squared residual (SRMR) [22]. Though the chi-squared test has been popularly used as a goodness of fit index, it has been reported that it is biased and usually not reliable as the only goodness of fit index. It is highly sensitive to sample size [23,24], and it is also often inflated by non-normally distributed data, which is often the case with physical activity data. To avoid such biases we employed the Hu and Bentler’s Two-Index Presentation Strategy combination rule to determine the best fit [22]. To determine the proportion of the total effect the absolute values for all indirect and direct effects were used.

## 3. Results

### 3.1. Descriptive Characteristics

The descriptive characteristics of the urban (*n* = 492) and rural (*n* = 476) participants are presented in Table 1. The urban girls were significantly older (mean 1.5 years) than the rural girls, and although BMI was not significantly different between the groups, the prevalence of overweight and obesity was higher in the urban compared to the rural group (46.5% vs. 38.8%). Waist circumference was not significantly different between the groups. SES was higher in the urban group, as the household SES index was significantly higher in the urban, compared to the rural, women (*p* < 0.001).

### 3.2. Differences in Physical Activity between the Urban and Rural Groups

**Figure 1:**
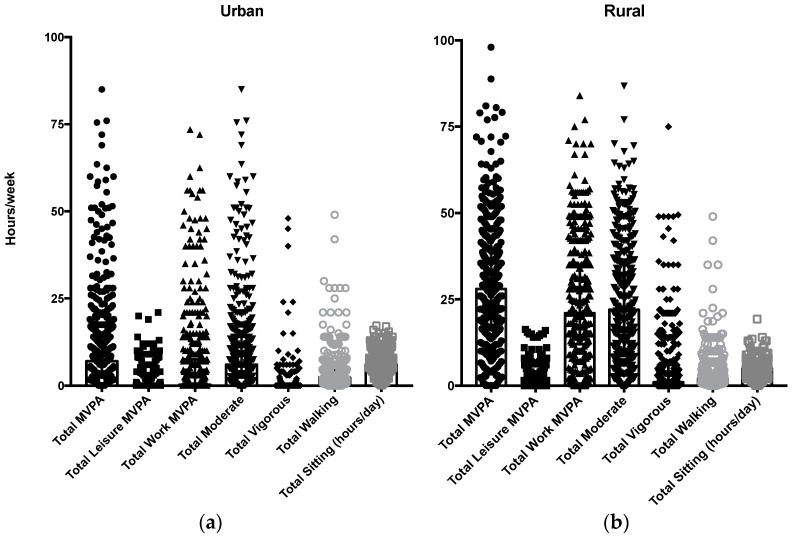
Comparative diagram of cumulative weekly physical activity for the different physical activity domains between South African urban (**a**) and rural (**b**) young adult women.

Self-reported physical activity levels in urban and rural girls are presented in Table 2. Rural girls were significantly more active than urban girls (*p* < 0.001), spending on average 1260 min more per week in MVPA. The majority of weekly MVPA was accumulated as occupational physical activity (an average of 21 h per week for rural girls vs. 45 min per week for urban girls, *p* < 0.001), and although more rural girls reported participating in leisure time MVPA than urban girls (48% vs. 22%); in those who participated, time spent in leisure time MVPA was higher in urban, than rural, girls (3 h 53 min vs. 2 h 8 min per week, *p* < 0.001). Urban girls spent, on average, 20 min more walking for travel per day than the rural girls (*p* = 0.06), but also 1 h more sitting per day (6 h vs. 5 h per day, *p* < 0.001). The distributions of these domains of activity for urban and rural girls are presented in Figure 1. There was a greater spread of time spent in activities such as total MVPA, total work MVPA and vigorous intensity physical activity in the rural women, while there was a greater concentration around the lower thresholds of time spent being active in the urban women. The converse is true for leisure time MVPA, where rural women showed a concentration around very small amounts of time in leisure time MVPA, while urban women showed a greater distribution of time spent in leisure time MVPA.

### 3.3. Structural Equation Models for BMI and Waist Circumference

The SEMs for the urban and rural samples separately, and for the combined sample on BMI, are presented in Table 3 (MVPA) and Table 4 (sitting time). The SEM of SES and MVPA on BMI in the urban sample showed that SES had a direct effect on MVPA, but was not associated with BMI. In the rural sample, SES had a direct effect on BMI that was not via MVPA, and there was no association between total household assets and MVPA. In the pooled sample, there was a significant direct effect of MVPA on BMI (0.04; 95% CI: 0.01; 0.08). The SEM model for SES, sitting time and BMI is presented in Table 4 for urban and rural girls separately, and then for the combined sample. In the urban sample there were no effects of SES on BMI either directly, or indirectly via time spent sitting. As shown in the MVPA model, in the rural girls there was a direct effect of SES on BMI, while in the pooled sample SES had a direct effect on time spent sitting, but this was not associated with BMI.

The SEM models for waist circumference are presented in Supplementary Table S1 (MVPA) and Supplementary Table S2 (sitting time) for the urban and rural groups separately and combined. These models showed similar results to the BMI models, where SES had a direct effect on MVPA (−4121; 95% CI: −72.85; −9.56) but no effect on waist circumference either directly or indirectly via MVPA, in the urban girls. In the rural sample, SES had a direct effect on waist circumference (0.62; 95% CI: 0.01; 1.23), with no significant effect on MVPA. In the combined sample, the same associations were evident as with BMI in the previous model. The model for SES, sitting time and waist circumference in the urban and rural girls, separately and combined (Supplementary Table S2), showed the same associations as were shown with BMI.

## 4. Discussion

The results of this study have shown that the relationship between SES (as measured by a sum of household assets) and lifestyle factors, BMI, and waist circumference, is different in urban compared to rural girls, suggesting that behavioural interventions need to be tailored to different communities. We have shown that while SES is associated with MVPA in urban women, it is associated with BMI in rural women, thereby illustrating that the results of economic transition may be different in communities at different stages of this process. This study highlights the direct and indirect effects of socio-economic and lifestyle factors on whole body and central adiposity, using structural equation modelling.

Although our results support previous studies [25,26,27], as well as the 2013 SANHANES data [28], which have shown a higher prevalence of overweight and obesity in urban compared to rural women of various ages, the margin of difference appears to be decreasing as the prevalence in rural groups increases. Nonetheless, prevalence of overweight and obesity is increasing in both groups, as evident from comparisons to a similarly aged (15–24 years) cohort of young black South Africans from 2003 in whom a prevalence of overweight/obesity of 33.9% and 25.7% in urban and rural women, respectively, was reported [26]. In contrast, the present study, conducted approximately 10 years later, reports a higher prevalence of overweight/obesity of 46.5% and 38.8% in urban and rural women, respectively.

Similar to other studies nationally and internationally, this study showed significant differences in physical activity volume and patterns between the urban and rural groups [25,26,29,30]. Time spent in moderate to vigorous intensity physical activity was nearly four times higher in the rural women, which was largely due to the contribution of occupational physical activity, as has been shown in other African countries [31]. In the Global Physical Activity questionnaire, occupational physical activity includes paid and unpaid work, as well as activities such as household chores. Time spent in the different physical activity domains showed greater distribution in the rural women, while there was more clustering in the lower ranges of time spent in physical activity in the urban women. The majority of urban (77%) and rural (97%) women were meeting the physical activity recommendations of 150 min of MVPA per week, however, sitting time was extremely high (average of 5 h a day), and less than half the women at both sites were participating in leisure time activity. It has been consistently reported that participation in leisure-time physical activity is low in African settings [31,32], possibly due to limited resources and opportunities. An unexpected finding was that time spent walking for travel was not different between the groups, however, this may be due to improved transport facilities and infrastructure in the rural villages in South Africa where, previously, people had to walk long distances [33]. Approximately 2 h of walking per week was reported in the rural women, which is less than a younger adolescent sample (11–15 years of age) from the same community who reported an average of more than 3 h of walking per week [2]. This may also be due to young adult lifestyles incorporating less routine driven activity, as the women in the present study were no longer in school or may not have been employed.

In the urban women as well as the combined sample, having a greater number of household assets, which is a well-recognised proxy for SES, was associated with less time spent in total MVPA. The association between SES and physical activity has been explored extensively, and may be different in low middle income countries (LMICs) compared to high income countries (HICs), which is often due to whether the predominant physical activity is discretionary or not [25,34,35,36]. We have previously shown a positive association between SES and MVPA in rural adolescents [2], however, in that study MVPA consisted only of time spent in discretionary activities i.e., school and club activities, while MVPA in the current study is a combination of all domains, which includes discretionary and non-discretionary physical activity. Interestingly, we did not see this association in the rural women which could be due to greater variance in SES in the urban women. Consistent with findings in other developing countries [1,37,38], this study reported a significant association between SES and sitting time. This association was found in the combined sample of urban and rural young adult women, and is the same as our previous results in a sample of rural South African adolescents [2], however, it differs to the findings from HICs [39,40,41]. In their systematic review and meta-analysis, Mielke et al., reported that in HICs, SES was inversely associated with sedentary behaviour (ES 0.67; 95% CI 0.62–0.73), whereas in low-middle-income countries, there was a positive association between SES and sedentary behaviour (ES 1.18; 95% CI 1.04–1.34).

The positive association between moderate-vigorous intensity physical activity and BMI, although contradictory to much of the literature [42,43], may be explained by factors associated with a community in transition. The increasing prevalence of overweight and obesity with increasing socio-economic status in countries undergoing epidemiological and nutrition transitions has been well described [44]. Changing dietary patterns and other environmental factors may make a more significant contribution to increasing overweight and obesity in transitioning communities [12], and these may occur in parallel with high levels of physical activity. The prevalence of overweight and obesity in the combined sample of young adult girls in the current study was 42.6%, however, they still reported participating in high levels of physical activity, equivalent to approximately 1192 min/week of MVPA (nearly three hours per day). Furthermore, 87% of participants were meeting physical activity guidelines. Physical activity is a behavioural measure at one time point, and although evidence suggests that physical activity tracks through childhood and adolescence, we have previously shown, convincingly, that young adult adiposity is associated with rate of progression through puberty, and with adiposity accumulation through childhood and adolescence [45]. Therefore, caution must be taken when interpreting these cross-sectional findings, as it is likely that other confounding factors besides those measured may be contributing to the associations presented.

This study does have some limitations, which includes the cross-sectional design. Although the urban and rural cohorts were from two different studies, both were conducted by the same research unit and, therefore, the methodology was harmonized between the two sites, thereby allowing for accurate comparison. These methods included clinical measures of general (BMI) and central (WC) adiposity, and although self-report measures of physical activity and sedentary behaviour were used, these methods have been validated in similar populations.

## 5. Conclusions

In conclusion, this study has shown that, despite high levels of physical activity reported in young adult women from rural and urban South Africa, overweight and obesity prevalence is still high. The influence of socio-economic status, and the role of physical activity and sedentary time, on whole body and central adiposity differs between the urban and rural samples, and the importance of other environmental and behavioural factors in the development of adiposity must be considered. Taking these factors into account when developing and designing interventions that target this vulnerable age group who are entering adulthood is critical for reducing the population burden of non-communicable disease, as well as future risk, not only for the individual, but also their offspring.

## Figures and Tables

**Table 1:** Descriptive characteristics of South African rural and urban young adult women.

Characteristic	Total	*n*	Urban	*n*	Rural	*p* Value
Age (years)	22.04 (1.24)	492	22.77 (0.49)	476	21.28 (1.31)	0.001
Weight (kg)	64.62 (14.82)	493	64.67 (15.6)	473	64.55 (14.03)	0.90
Height (m)	1.61 (0.007)	492	1.60 (0.07)	475	1.61 (0.07)	0.001
BMI (kg/m^2^)	25.05 (5.59)	492	25.32 (5.91)	473	24.78 (5.24)	0.13
BMI classification (%)						0.015
Underweight (<18.4 kg/m^2^)	5.98		7.10		4.82	
Normal weight (18.5–24.9 kg/m^2^)	51.34		46.45		56.39	
Overweight (25–29.9 kg/m^2^)	26.19		29.21		23.06	
Obese (>30 kg/m^2^)	16.49		17.24		15.72	
Waist circumference (cm)	80.60 (12.08)	493	80.18 (12.63)	477	81.03 (11.47)	0.26
Household SES index (sum of assets)	7.24 (2.70)	493	8.83 (2.37)	476	5.59 (1.91)	0.001
Highest Education attained (%)		480		371		0.001
Primary school	1.18		0.00		2.70	
Secondary school	60.75		48.33		76.82	
Tertiary education	38.07		51.67		20.49	

Continuous data presented as mean (SD) and categorical data presented as %.

**Table 2:** Self-reported physical activity domains (minutes/week) of South African urban and rural young adult women.

Physical Activity Domain	Total Median (IQR)	*n*	Urban Median (IQR)	*n*	Rural Median (IQR)	*p* Value
Total MVPA (minutes/week)	870 (280–1810)	492	420 (160–900)	385	1680 (970–2580)	<0.001
Total leisure time MVPA (minutes/week)	0 (0–90)	492	0 (0–0)	385	0 (0–120)	<0.001
(Excluding Zero)	180 (90–360)	110	233 (120–360)	184	128 (60–290)	<0.001
Total work MVPA (minutes/week)	450 (0–1400)	484	45 (0–450)	385	1260 (720–2100)	<0.001
Total moderate PA (minutes/week)	630 (210–1550)	492	360 (140–840)	385	1320 (525–2190)	<0.001
Total vigorous PA (minutes/week)	0 (0–90)	492	0 (0–0)	385	60 (0–360)	<0.001
Total walking for travel (minutes/week)	120 (60–250)	488	140 (65–275)	385	120 (60–240)	0.060
Sitting time (minutes/day)	300 (240–480)	492	360 (240–480)	385	300 (180–360)	<0.001

MVPA—moderate to vigorous physical activity, PA—physical activity.

**Table 3:** Structural equation models for SES (sum of household assets) and moderate-vigorous intensity physical activity on BMI in South African urban and rural young adult women, separately and pooled.

Effect of:	Outcome:	Direct Effects (95% CI)	Indirect Effects (95% CI)	Total Effects (95% CI)	Proportion of Total Effect Mediated
Household assets (urban)	BMI	0.14 (−0.09; 0.36)	−0.015 (−0.043; 0.013)	0 .121 (−0.101; 0.343)	0.1 ^a^
	via MVPA				
	MVPA	−41.69 (−73.40; −9.98) **		−41.69 (−73.40; −9.98) **	
MVPA (urban) ^#^	BMI	0.04 (−0.03; 0.1)		0.04 (−0.03; 0.1)	
Household assets (rural)	BMI	0.306 (0.03; 0.59) *	−0.009 (−0.033; 0.014)	0.30 (0.02; 0.58) *	0.03 ^a^
	via MVPA				
	MVPA	−30.33 (−88.42; 27.76)		−30.33 (−88.42; 27.76)	
MVPA (rural) ^#^	BMI	0.03 (−0.02; 0.08)		0.03 (−0.02; 0.08)	
Household assets (pooled)	BMI	0.14 (−0.011; 0.30)	−0.06 (−0.113; −0.006) *	0.083 (−0.07; 0.23)	0.3 ^a^
	via MVPA				
	MVPA	−144 (−170.34; −119.04) ***		−144 (−170.34; −119.04) ***	
MVPA (pooled) ^#^	BMI	0.04 (0.01; 0.08) *		0.04 (0.01; 0.08) *	

Adjusted for age; * *p* < 0.05; ** *p* < 0.01; *** *p* < 0.001; ^a^ Assessed using the absolute values for both indirect and direct effects. ^#^ MVPA multiplied by 100. MVPA; moderate to vigorous physical activity, BMI; body mass index. Urban Fit Indices: LR test of model vs. saturated: chi2(2) = 0.99, Prob > chi2 = 0.63; RMSEA = 0.00; CFI = 1.00 Comparative fit index; TLI = 1.53 Tucker-Lewis index; SRMR = 0.011: Standardized root mean squared residual, CD = 0.017 Coefficient of determination. Rural Fit Indices: LR test of model vs. saturated: chi2(2) = 1.37, Prob > chi2 = 0.50; RMSEA = 0.00; TLI = 1.14 Tucker-Lewis index; SRMR = 0.02: Standardized root mean squared residual, CD = 0.03 Coefficient of determination. Pooled Fit Indices: LR test of model vs. saturated: chi2(2) = 18.61, Prob > chi2 = 0.000; RMSEA = 0.098; CFI= 0.88 Comparative fit index; TLI = 0.71 Tucker-Lewis index; SRMR = 0.034: Standardized root mean squared residual, CD = 0.135 Coefficient of determination.

**Table 4:** Structural equation model for SES (sum of household assets) and sitting time on BMI in South African urban and rural young adult women, separately and pooled.

Effect of:	Outcome:	Direct Effects (95% CI)	Indirect Effects (95% CI)	Total Effects (95% CI)	Proportion of Total Effect Mediated
Household assets (urban)	BMI	0.121 (−0.101; 0.3433)	−0.0003 (−0.0151; 0.0145)	0.12 (−0.10; 0.34)	0.002 ^a^
	via sitting time				
	Sitting time	38.77 (−12.39; 89.92)		38.77 (−12.39; 89.92)	
Sitting (urban)	BMI	0.00 (−0.0003; 0.0004)		0.00 (−0.0003; 0.0004)	
Household assets (rural)	BMI	0.30 (0.02; 0.58) *	−0.002 (−0.02; 0.017)	0.298 (0.02; 0.58) *	0.01 ^a^
	via sitting time				
	Sitting time	38.24 (−21.37; 97.85)		38.24 (−21.37; 97.85)	
Sitting (rural)	BMI	−0.000 (−0.0005; 0.0004)		−0.000 (−0.0005; 0.0004)	
Household assets (pooled)	BMI	0.10 (−0.05; 0.25)	−0.006 (−0.036; 0.0234)	0.090 (−0.06; 0.24)	0.06 ^a^
	via sitting time				
	Sitting time	101.45 (69.75; 133.15) ***		101.45 (69.75; 133.15) ***	
Sitting (pooled)	BMI	−0.000 (−0.0004; 0.0002)		−0.000 (−0.0004; 0.0002)	

Adjusted for age; * *p* < 0.05; ** *p* < 0.01; *** *p* < 0.001; ^a^ Assessed using the absolute values indirect and direct effects. BMI; body mass index. Urban Fit Indices: LR test of model vs. saturated: chi2(2) = 1.91, Prob > chi2 = 0.38; RMSEA = 0.00; CFI = 1.00 Comparative fit index; TLI = 1.47 Tucker-Lewis index; SRMR = 0.017: Standardized root mean squared residual, CD = 0.007 Coefficient of determination. Rural Fit Indices: LR test of model vs. saturated: chi2(2) = 0.023, Prob > chi2 = 0.989; RMSEA = 0.00; TLI = 1.56 Tucker-Lewis index; SRMR = 0.002: Standardized root mean squared residual, CD = 0.035 Coefficient of determination. Pooled Fit Indices: LR test of model vs. saturated: chi2(2) = 10.66 Prob > chi2 = 0.005; RMSEA = 0.070; CFI = 0.834 Comparative fit index; TLI = 0.59 Tucker-Lewis index; SRMR = 0.027: Standardized root mean squared residual, CD = 0.052 Coefficient of determination.

## Supplementary Materials

The following are available online at www.mdpi.com/1660-4601/14/10/1271/s1.
