# Supplementary material for: Understanding the Relationship between Socio-Economic Status, Physical Activity and Sedentary Behaviour, and Adiposity in Young Adult South African Women Using Structural Equation Modelling
Source: Int J Environ Res Public Health. 2017 Oct 23;14(10):1271. doi: 10.3390/ijerph14101271 (PMC5664772; doi:10.3390/ijerph14101271)
Supplement: Supplementary file 1 [file ijerph-14-01271-s001.pdf]

International Journal of *Environmental Research and Public Health*

MDPI

Supplementary Materials

Understanding the Relationship between Socio-Economic Status, Physical Activity and Sedentary Behaviour, and Adiposity in Young Adult South African Women Using Structural Equation Modelling

Lisa K. Micklesfield, Richard J. Munthali[1], Alessandra Prioreschi, Rihlat Said-Mohamed, Alastair van Heerden, Stephen Tollman, Kathleen Kahn, David Dunger and Shane A. Norris

Table S1. Structural equation model for household assets and moderate-vigorous intensity physical activity on waist circumference in South African urban and rural young adult women, separately and pooled.

Effect of:	Outcome:	Direct effects (95% CI)	Indirect effects (95% CI)	Total effects (95% CI)	Proportion of total effect mediated
Household assets (urban)	WC	−0.14 (−0.61; 0.34)		−0.17 (−0.64; 0.31)	0.18
	via MVPA		−0.030 (−0.09; 0.028)		
	MVPA	−41.21 (−72.85; −9.56) **		−41.21 (−72.85; −9.56) **	
MVPA (urban) #	WC	0.0007 (−0.001; 0.002)		0.0007 (−0.001; 0.002)	
Household assets (rural)	WC	0.62 (0.01; 1.23) *		0.604 (−0.01; 1.21) *	0.03 [a]
	via MVPA		−0.016 (−0.060; 0.028)		
	MVPA	−27.35 (−84.96; 30.27)		−27.35 (−84.96; 30.27)	
MVPA (rural) #	WC	0.001 (−0.0005; 0.0016)		0.001 (−0.001; 0.002)	
Household assets (pooled)	WC	−0.1 (−0.43; 0.23)		−0.25 (−0.57; 0.07)	0.6
	via MVPA		−0.15 (−0.267; −0.037) **		
	MVPA	−142.73 (−168.27; −117.20) ***		−142.73 (−168.27; −117.20) ***	
MVPA (pooled) #	WC	0.001 (0.0003; 0.0018) **		0.001 (0.0003; 0.0018) **	

Adjusted for age; * $p < 0.05$; ** $p < 0.01$; *** $p < 0.001$; [a] Assessed using the absolute values for both indirect and direct effects. # MVPA multiplied by 100. MVPA; moderate to vigorous physical activity, WC; waist circumference. Urban Fit Indices: LR test of model vs. saturated: chi2(2) = 0.896, Prob > chi2 = 0.639; RMSEA = 0.00; CFI = 1.00. Comparative fit index; TLI= 1.37 Tucker-Lewis index; SRMR = 0.011: Standardized root mean squared residual, CD= 0.020 Coefficient of determination. Rural Fit Indices: LR test of model vs. saturated: chi2(2) = 1.46, Prob > chi2 = 0.48; RMSEA = 0.00; TLI= 1.12 Tucker-Lewis index; SRMR = 0.017: Standardized root mean squared residual,

CD = 0.035 Coefficient of determination. Pooled Fit Indices: LR test of model vs. saturated: chi2(2) = 18.25, Prob > chi2 = 0.000; RMSEA = 0.096; CFI = 0.88 Comparative fit index; TLI= 0.70 Tucker-Lewis index; SRMR = 0.034: Standardized root mean squared residual, CD = 0.126 Coefficient of determination.

Table S2. Structural equation model for household assets and sitting time on waist circumference in South African urban and rural young adult women, separately and pooled.

Effect of:	Outcome:	Direct effects (95% CI)	Indirect effects (95% CI)	Total effects (95% CI)	Proportion of total effect mediated
Household assets (urban)	WC	−0.17 (−0.64; 0.31)		−0.172 (−0.640; 0.304)	0.01
	via sitting time		−0.002 (0.033; 0.029)		
	Sitting time	37.53 (−13.54; 88.59)		37.53 (−13.54; 88.59)	
Sitting time (urban)	WC	−0.000 (−0.0009; 0.0008)		−0.000 (−0.0009; 0.0008)	
Household assets (rural)	WC	0.602 (−0.008; 1.21) *		0.605 (−0.004; 1.215) *	0.005
	via sitting time		0.003 (−0.027; 0.034)		
	Sitting time	28.65 (−30.59; 88.00)		28.65 (−30.59; 88.00)	
Sitting time (rural)	WC	0.0001 (−0.001; 0.001)		0.0001 (−0.001; 0.001)	
Household assets (pooled)	WC	−0.217 (−0.542; 0.107)		−0.232 (−0.553; 0.088)	0.06
	via sitting time		−0.0147 (−0.077; 0.048)		
	Sitting time	98.20 (66.60; 129.81) ***		98.20 (66.60; 129.81) ***	
Sitting time (pooled)	WC	−0.0001 (−0.0008; 0.0005)		−0.0001 (−0.0008; 0.0005)	

Adjusted for age; * $p < 0.05$; ** $p < 0.01$; *** $p < 0.001$; [a] Assessed using the absolute values for both indirect and direct effects. WC; waist circumference. Urban Fit Indices: LR test of model vs. saturated: chi2(2) = 1.97, Prob > chi2 = 0.37; RMSEA = 0.00; CFI = 1.00 Comparative fit index; TLI= 1.027 Tucker-Lewis index; SRMR = 0.017: Standardized root mean squared residual, CD = 0.012 Coefficient of determination. Rural Fit Indices: LR test of model vs. saturated: chi2(2) = 0.00, Prob > chi2 = 1.00; RMSEA = 0.00; TLI = 1.55 Tucker-Lewis index; SRMR = 0.000: Standardized root mean squared residual, CD = 0.036 Coefficient of determination. Total Fit Indices: LR test of model vs. saturated: chi2(2) = 10.31 Prob > chi2 = 0.006; RMSEA = 0.069; CFI = 0.82 Comparative fit index; TLI = 0.554 Tucker-Lewis index; SRMR = 0.027: Standardized root mean squared residual, CD = 0.046 Coefficient of determination.
